# Harnessing Exosomes: A Brief Overview of Nature’s Nanocarriers and a Glimpse into Their Implications in Pituitary Neuroendocrine Tumors (PitNETs)

**DOI:** 10.3390/cimb47050310

**Published:** 2025-04-28

**Authors:** Ligia Gabriela Tataranu

**Affiliations:** 1 Department of Neurosurgery, Carol Davila University of Medicine and Pharmacy, 020021 Bucharest, Romania; ligia.tataranu@umfcd.ro; 2 Department of Neurosurgery, Bagdasar-Arseni Emergency Clinical Hospital, 041915 Bucharest, Romania

**Keywords:** extracellular vesicles, exosomes, nanocarriers, PitNETs, biomolecular

## Abstract

The study of exosomes is currently an area of major interest in the scientific world, especially after the discovery of their function as natural nanocarriers. Their intrinsic features in regulating intricate intracellular pathways have put them in the spotlight in the last decade, and it has been considered that by harnessing them, the future of cellular communication and therapeutic innovation will experience a breakthrough, leading to pioneering research. However, it has been demonstrated that exosomes have various important roles, from conferring resistance to viral infections of the human placenta to transfer of oncogenic signals between cells, reshaping cellular metabolism, promoting angiogenesis, mediating immune evasion, serving as biomarkers in cancer diagnosis and prognosis, and having implications in the therapeutic management of certain diseases. Besides the general overview of exosomes as nature’s nanocarriers and their functions, this article aims to discuss their implications in PitNETs, especially since there have been many recent studies regarding the clinical benefits of biomolecular medicine.

## 1. Introduction

Extracellular vesicle (EV) is used as a generic name to refer to secreted vesicles and represents an element released by all living cells, prokaryotes, and eukaryotes as part of their normal life cycle or after the occurrence of an anomaly [1]. The EVs are lipid bilayer membrane structures whose main activity is to carry bioactive molecules through the extracellular spaces, and it is worth mentioning that these elements reflect the features of the originating cells [2]. The two main categories of EVs are ectosomes and exosomes. The ectosomes are components that bud directly from the cell surface, and their main activities are represented by the expression of phosphatidylserine and anti-inflammatory/immunosuppressive features [3]. Thus, while ectosomes are shed by cells by budding from their membrane, the exosomes are the opposite result and are formed by inward budding into the endosomal cavity [3].

**Figure 1:**
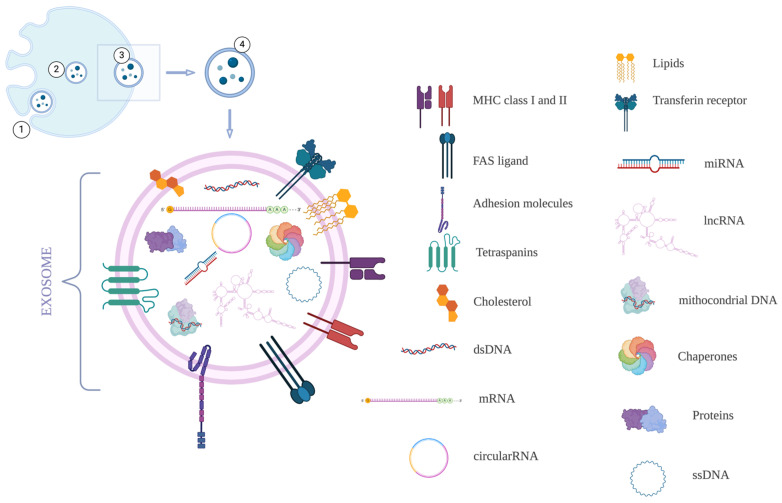
The biogenesis and contents of an exosome: 1—Endocytic vesicle in a parental cell; 2—Endosome; 3—MVB with ILVs; 4—Exosome. Elaborated with BioRender (individual).

Exosomes are EVs with a diameter of less than 150 nm that arise from the endosomal pathway. The cellular membrane protrudes inward, filling its luminal part with accumulating intraluminal vesicles (ILVs), the future exosomes. These ILVs are contained in a multivesicular body (MVB) [4]. Through fusion with the plasma membrane and exocytosis, ILVs are secreted as exosomes. These exosomes contain all cellular molecular elements, such as proteins, RNA, and DNA. Furthermore, following multiple invaginations, the orientations of the lipid bilayer of exosomes reflect that of the originating cell [5]. However, the exosomes are not only composed of a lipid bilayer. These small vesicles also contain cytosolic proteins, DNA, messenger RNA, and small non-coding RNA. They are present in all body fluids, expressing biomarkers, making them perfect candidates for minimally invasive or non-invasive diagnosis (Figure 1). Moreover, by helping to transfer various biomolecular elements inside targeted cells, exosomes are considered major intercellular communicators and powerful therapeutics [6]. Currently, several exosomal components can provide important information regarding the biomolecular state of the originating cell, and their contents can be harnessed to diagnose multiple diseases, as major classes of molecular cargo can be used as biomarkers [7].

A comprehensive and intricate article regarding the prospective roles of exosomes in pituitary tumors has been recently published by Paulina Lisiewicz, Małgorzata Szelachowska, Adam Jacek Kretowski, and Katarzyna Siewk [8]. Their article represents a cornerstone that focuses on exosomes in PitNETs, whereas the current article refers mainly to exosomes in general, with just a glimpse into the pathology of PitNET.

Regarding the major roles of exosomes, besides their crucial involvement in intercellular communication by carrying proteins, nucleic acids, and metabolites, it has been stated that they are involved in immunity and metastasis, and they support tumor formation by contributing to angiogenesis. Furthermore, they can dramatically alter the tumor microenvironment, supporting tumoral cell survival, immune evasion, and drug resistance. Therefore, given their complexity, circulating exosomes are used as liquid biopsies and biomarkers for the prompt detection, diagnosis, and treatment of many diseases, especially cancer [9].

## 2. Roles of Exosomes

The last decade has shed more light on the functions and implications of exosomes in vivo and in vitro. While some functions are already established, others are still referred to as possibilities and are still being researched. Further studies are encouraged to develop this promising field of interest in biomolecular medicine. A summary of the most important roles of exosomes which will be discussed further:

1. Roles in infection and pregnancy
2. Transfer of oncogenic signals between cells
3. Communication between neoplastic cells and the tumor microenvironment
4. Reshaping cellular metabolism
5. Promoting angiogenesis
6. Mediating immune evasion
7. Biomarkers for diagnosis and prognosis
8. Drug delivery vehicles
9. Therapeutic target

### 2.1. Roles in Infection and Pregnancy

It has been demonstrated that exosome-mediated delivery of specific microRNAs confers resistance to viral infections of the human placenta [10]. Primary human trophoblast-derived exosomes have a direct role in the transfer of viral resistance to non-placental recipient cells. They induce autophagy and protect against viral infections with herpes simplex virus 1, poliovirus, and human cytomegalovirus [10]. Furthermore, it has been stated that the exosomal microRNA profile can change across gestation. Thus, it can be used as a biomolecular marker of the progression of the pregnancy [11]. Sheller-Miller et al. concluded that exosomes can function as labor and delivery paracrine mediators. The authors demonstrated this paracrine communication between fetal and maternal tissues through exosomes and found that exosomes promote proinflammatory processes to prepare the cervix and uterus for parturition. Besides these findings, the study also reported that although the size and shape of the exosomes remained constant throughout the pregnancy, their quantity increased with gestational age and returned to normal within a week postpartum [12]. In clinical practice, changes in the exosome profile could be used to diagnose placental dysfunctions [13].

### 2.2. Transfer of Oncogenic Signals Between Cells

In healthy tissues, communication between cells is crucial to maintain physiological homeostasis. Besides the direct contact between these cells, there is another form of communication, through exosomes [14]. It has been demonstrated that these elements are involved not only in local and distant intercellular communication but also in intracellular communication [14].

In cancer, while oncosomes can carry many more tumor-derived molecules given their more significant volumes, the role of exosomes is still major, notwithstanding their smaller size [15]. Intercellular communication through exosomes is necessary for remodeling the tumor microenvironment during tumorigenesis. However, their importance is not only limited to this remodeling, as exosome-mediated communication is also required to form premetastatic niches [16]. Furthermore, the exosomes originating from neoplastic cells contain bioactive molecules that are mandatory in reprogramming cells and the architecture in tumor microenvironments or premetastatic niches [16]. By providing these bioactive molecules, the exosome supports and influences tumoral cell division and survival [17,18,19]. In a recent article regarding exosome-mediated intercellular transfer, Wu et al. concluded that exosomes confer resistance to chemotherapy and radiotherapy to non-resistant cancerous cells in vivo and in vitro through horizontal transfer. The authors stated that targeting these exosomes may prevent or even reverse this resistance to these therapeutic options in the future [20]. However, it has been discovered that not only can the resistance to therapy be transferred through these small extracellular vesicles, as Jin et al. demonstrated that exosomes originating from a highly malignant pancreatic cell line named PC-1.0 can enhance proliferation, migration, and invasion abilities in a moderately malignant pancreatic cell line named PC-1 [21].

### 2.3. Communication Between Neoplastic Cells and the Tumor Microenvironment

In neoplastic diseases, exosomes are a way of communication between healthy and cancerous cells. This is especially the case for cancer-associated fibroblasts. Webber et al. demonstrated that exosomes from cancerous cells can drive several changes in normal fibroblasts through TGF-β1, transforming them into cancerous cells [22]. Furthermore, the authors revealed that exosomes with low levels of betaglycan expression will exhibit low levels of TGF-β and are not able to induce changes in healthy fibroblasts [22]. The study suggests that in terms of biological function, the impact of cancer exosomes on primary fibroblasts is significant, as these exosomes are an additional mechanism that contributes to the modulation of the stromal-extracellular matrix as a consequence of fibroblast differentiation [22]. While Ringuette et al. concluded the same in their article [23], other authors discovered that fibroblast transformation is possible through the transfer of microRNA-125b [24] or microRNA-1247-3p. Tumor-derived exosomes can induce cancerous cell proliferation through phosphatidylinositol 3-kinase/protein kinase B (PI3K/AKT) and MAPK/ERK signaling pathways. Furthermore, these exosomes derived from tumoral cells can enhance the migratory capacity of the tumor recipient cells, promoting and supporting invasiveness and motility [25].

### 2.4. Reshaping Cellular Metabolism

Besides the aforementioned involvement of stromal cells, a mechanistic model involving them and the exosomes that induce a metabolic program has been described [26]. The exosomal microRNA-105 is caused by the oncoprotein MYC in cancerous cells. In favorable settings in the tumor microenvironment, cancer-associated fibroblasts increase glucose and glutamine through exosomes containing microRNA-105. In unfavorable settings, such as a lack of nutrients in the tumor microenvironment, microRNA-105 will induce a metabolic program in the fibroblasts to transform the waste into energy-rich metabolites. Consequently, the microRNA-105 metabolic reprogramming of cancer-associated fibroblasts contributes to sustained tumor growth by conditioning the shared metabolic environment [26].

Another study shows that cancer cell-derived exosomes contribute to the functional heterogeneity of activated fibroblasts by reprogramming their proteome [27]. The study also demonstrates that the fibroblasts activated by the exosomes in the early stages of the cancerous disease display a high amount of pro-angiogenic and pro-proliferative proteins and, consequently, they highly promote proliferation and angiogenesis. On the other hand, in the later stages of the disease, the fibroblasts have a greater capacity for invasion into the extracellular matrix [27].

The possibility of exosomal adrenomedullin involvement in the development of diabetes in pancreatic cancer has also been studied in the last decade. It has been stated that the lipolysis-inducing cargo is carried in exosomes originating from pancreatic cancer cells and is responsible for paraneoplastic effects such as new-onset diabetes and weight loss before the clinical presentation of cancer [28]. Similar to these findings, it has been shown that cancer-derived exosomes can induce lipolysis in adipocytes, while the lipolysis can be inhibited using the neutral sphingomyelinase inhibitor GW4869 [29].

These findings suggest that exosomes originating from cancer cells can impact distant cells’ metabolism in many ways, promoting tumoral growth, proliferation, and metastasis [30].

### 2.5. Promoting Angiogenesis

Concerning angiogenesis, many studies have demonstrated the involvement and major roles of exosomes and other extracellular vesicles in preclinical and clinical settings. Taverna et al. offered insights into the roles of exosomes in angiogenesis stimulated by chronic myelogenous leukemia cells, concluding that these specific extracellular vesicles released by LAMA84 CML cells can affect angiogenesis in vitro and in vivo [31]. The entire process is modulated by these exosomes, enriched in Vascular Cell Adhesion Molecule 1 and Intercellular Adhesion Molecule 1. Furthermore, the exosomes released near the endothelial cells may influence the exacerbation of endothelium activation and the following migration of endothelial cells during angiogenesis [31]. Umezu et al. discovered that microRNAs from the 17–92 cluster are responsible for regulating endothelial gene expression during tumor angiogenesis and have a major role in neoplasia-to-endothelial cell communication [32]. Moreover, it has been demonstrated that exosomes derived from human umbilical cord plasma promote angiogenesis and fibroblast function through microRNA-21-3p. Not only has this type of RNA been found to be highly enriched in exosomes derived from the human umbilical cord, but it also serves as an important mediator for regulatory effects through the inhibition of phosphatase and tensin homolog and sprouty homolog 1 [33].

It is essential to mention that exosomes from multiple myeloma contain proteins like VEGF, basic fibroblast growth factor, serpin E1, hepatocyte growth factor, and matrix metalloproteinase-9, which are considered major angiogenic factors [34]. Furthermore, multiple myeloma-derived exosomes are responsible for modulating the bone marrow microenvironment. Consequently, by enhancing angiogenesis and immunosuppression, these exosomes will further facilitate the progression of the disease [34].

The role of extracellular vesicles in angiogenesis has also been studied in human lung cancer A549, where a sortilin-containing complex exhibits control over endothelial cells and neovascularization through exosome transfer [35].

Another crucial element in modulating the communication between tumoral and endothelial cells is represented by hypoxia. In some cancers, such as glioblastoma, the proteome and messenger RNA profiles of exosomes reflect the oxygenation status of glioma cells and patient tumors, while the exosomal pathway represents a targetable driver of hypoxia-dependent intercellular signaling during tumorigenesis [36]. If exosomes originating from brain cancer cells were developed during a hypoxic period, they become inducers of angiogenesis through phenotypic modulation of endothelial cells [36]. Besides that, it has been demonstrated that in the same hypoxic settings, proteins with major roles in angiogenesis are highly transported by exosomes, even in other types of cancers, such as nasopharyngeal, prostate, and ovarian cancer [37,38,39,40]. Moreover, in some cases, these elements and mechanisms not only induce angiogenesis but also facilitate metastasis [41].

### 2.6. Mediating Immune Evasion

The immune system recognizes cancerous cells as hostile. Notwithstanding this, in many circumstances, these cells can develop the ability to avoid or block it, even in a normal host immune system. Immune evasion represents a significant obstacle to effective therapeutic approaches in cancer diseases [42].

The role of exosomes in the dynamic interaction between cancer cells and the immune system enables them to mediate various processes associated with metastasis. For example, exosomal circCOG2 released by cancer cells in normoxic conditions can regulate these cells’ invasion and migration capabilities. This happens by activating a signaling pathway called microRNA-1305/GF-β2/SMAD3 [43].

Exosomes originating from tumors impact the immune system through programmed death ligand 1 (PD-L1), transforming growth factor-beta (TGF-beta), Fas cell surface death receptor ligand (FasL), and other proteins to cause immunosuppression [44,45]. Furthermore, exosomes can mediate immunosuppression by activating immune checkpoint signaling and T-cell dysfunction. This occurs via direct binding of PD-L1 secreted in exosomes to its receptor PD1 expressed on the surface of activated T cells [44].

Exosomes as fatty acid carriers have been demonstrated to prompt a metabolic shift towards oxidative phosphorylation, driving dendritic cell immune impairment. However, transcriptomic data identify peroxisome proliferator-activated receptor alpha as a fatty acid sensor regulating the immunosuppressive effects on dendritic cells. The peroxisome proliferator-activated receptor alpha inhibition restores the function of dendritic cells and consequently increases the effectiveness of immunotherapy [45].

In glioma, the circular RNA of exon-encoded origin by Nei Like DNA Glycosylase 3 (circNEIL3) is packaged into exosomes by hnRNPA2B1 and transmitted to infiltrated tumor-associated macrophages, conferring the immunosuppressive ability, which will support and promote tumor progression [46,47].

While increasing evidence suggests the important role of exosomes as mediators of immune regulation [48,49,50,51], it is worth mentioning that in some cases, these extracellular vesicles promote immune cell differentiation in a favorable way to the tumor. Not only can they induce regulatory T-cell production and upregulate their immunosuppressive role, but they can also disrupt the differentiation of monocytes to dendritic cells and promote the generation of myeloid immunosuppressive cells [52].

### 2.7. Biomarkers for Diagnosis and Prognosis

In the last decades, the presence of exosomes has been reported in most human biofluids, containing proteins, lipids, and microRNAs that can mirror not only the cellular origin but also its physiological state [53]. Thus, it has been concluded that besides other known functions, exosomes can be used as biomarkers for several diseases [53].

Tanaka et al. demonstrated that serum exosomes from patients with cancerous disease can induce proliferation in vitro. Furthermore, levels of microRNA-21 were very high in patients with cancer compared to patients with benign diseases, which highlights that exosomal microRNA-21 can be a clinical biomarker. To sustain these findings, the authors stated that after exosome extraction from the patient’s serum, no trace of microRNA-21 was detected. Besides, the study demonstrated that the expression of this type of exosomal RNA has been associated with aggressiveness and progression [54]. Similar results regarding the serum exosomal microRNA-21 were also reported by Wang et al. when the authors noted the significant difference between the amounts of microRNA found in the serum before and after exosome removal [55].

Four other types of biofluid-originating microRNA were discovered as associated with cancer, respectively 1246, 3976, 4306, and 4644, while two of them, 1246 and 4644, were considered eligible candidates as biomarkers for early-stage cancer diagnosis [56]. However, exosomes can not only be used as biomarkers in cancer diagnosis. Research studies demonstrated that exosomal transcription factors, which are considered a new class of biomarkers, can be used to diagnose renal diseases [57,58]. The urinary exosome aquaporin levels correlate with the level of apical membrane expression in renal tubules. The urinary exosomal aquaporins related to human kidney disease are aquaporins 1 (AQP1) and 2 (AQP2). Low levels of AQP1 are related to urinary tract obstruction, while low levels of AQP2 are related to urinary concentration defects [59]. Exosomal Fetuin-A has been identified by proteomics and represents a biomarker for acute kidney disease [60]. Urinary exosomal Renal Wilms’ tumor-1 is also a promising biomarker with podocyte specificity that can detect early progression and treatment-induced regression of podocyte injury [61].

It is worth mentioning that while exosomes originating from various cell types have similar biophysical characteristics and proteomic profiles, those originating from brain tumors have unique features [62]. Some of these features were described by Graner et al. in an article regarding the proteomic and immunologic analyses of brain tumor exosomes. The authors of this study mentioned the very basic isoelectric points and expressed the mutated tumor antigen EGFRvIII and the putatively immunosuppressive cytokine TGF-β as some of these unique features [62].

San Lucas et al. demonstrated high-resolution profiling of the genomic and transcriptomic landscapes of visceral cancers [63]. The authors stated that the short half-life of the DNA in the exosome allows for a very accurate depiction of the tumoral dynamics and gives the possibility of tracking the tumoral evolution, as well as its response to therapeutic management [63].

### 2.8. Drug Delivery Vehicles

As has already been mentioned, exosomes are involved in numerous biological activities, and evidence suggests that they can also be used as therapeutic targeted agents. The reasons behind these scientific conclusions are represented by their abilities to target specific cells, their ability to transfer genetic material, and their good host biocompatibility, which allows them to avoid being taken up by the macrophages and get through the extracellular matrix and vascular walls [64]. Yong et al. described five characteristics needed to achieve good anticancer activity after administering exosome-based drugs. These characteristics are represented by long circulation, an increased accumulation and penetration at the tumoral site, efficient internalization, and drug release [65].

Most targeted drugs address intracellular components; thus, they must travel across cellular membranes. When it comes to the loaded exosomes, it’s their lipid bilayer that protects the drug. Furthermore, the low level of toxicity and minimal immunogenicity of the exosome represent the main benefits since immune system clearance can be easily avoided [4]. For example, the incorporation of Paclitaxel in exosomes secreted by mesenchymal stromal cells has been proven beneficial for tumoral growth inhibition in vitro [66]. However, exosomes were reported to be beneficial as drug delivery vehicles in many cases in vivo and in vitro [67,68,69], even in diseases like hyperhomocysteinemia [70] or Parkinson’s disease [71].

Last but not least, it is important to mention that the technologies behind using exosomes as a form of drug delivery vehicles are represented by directly treating the exosomes themselves or by loading the exosomes secreted by the parent cell with drugs [72].

### 2.9. Therapeutic Target

Besides being drug delivery vehicles, exosomes themselves can be therapeutic targets. The goal of these therapeutic approaches is not only to reduce the production of exosomes but also to inhibit their secretion and uptake. Furthermore, these mechanisms aim to block intercellular and intracellular communication through exosomes, as well as to eliminate certain exosome components [4].

Since an important component of these extracellular vesicles is represented by the lipids, which are essential for their membranes, it could be a viable targeting element, as their reduction will lead to a decrease in exosome release [73].

Proteins are also an important membrane component and are considered rich in targets for therapeutic application. Proteomic evidence suggests that various proteins can be expressed on the surface of exosomes originating from cancer cells, which makes them great targets for clinical diagnosis or treatment. Usually, for membrane proteins to be useful as therapeutic methods, they must undergo different processes, such as solubilization. However, it is essential to mention that the most significant advantage of exosomes lies in their ability to offer a perfect membrane environment for membrane proteins. Thus, there is no need for additional solubilizing processes [74].

Nevertheless, genetic engineering and chemical modification are useful to enhance exosomal capabilities to target cells or tissues, to reduce the damage of drugs, as well as to reduce the toxicity and side effects of these drugs [75]. Besides, physical and chemical modifications of exosomes also represent common techniques for targeting them. For example, the preparation of magnetic exosomes by coating magnetic nanoparticles or covalent modification, and the use of ligand/receptor interaction, were reported methods [75].

However, despite the great potential of exosomes as targeted elements in the therapeutic approach to different diseases, there are also many challenges and shortcomings, such as the separation and purification, as well as their constantly changing nature [76]. For that reason, it is still difficult to implement the clinical use of targeting exosomes, and further research is needed [76].

## 3. Researched Implications of Exosomes in PitNETs

Pituitary neuroendocrine tumors (PitNETs) are typically benign intracranial lesions originating from the anterior hypophysis. Usually, these tumors present a broad spectrum of clinical manifestations that can be divided into two categories. One category comprises signs and symptoms of excessive hormone secretion, while the other includes manifestations resulting from the tumoral mass compression on the neighboring anatomical structures [77].

The availability of neuroimaging studies in the last decades has led to an increase in incidentally found PitNETs and clinically relevant ones [78]. The current incidence ranges between 3.9 and 7.4 cases per 100,000 yearly, and the prevalence varies between 76 and 116 cases per 100,000 in the general population. Most new cases are prolactinomas and nonsecreting PitNETs. A trend towards female predominance has been reported in clinically relevant pituitary lesions; however, PitNETs are clinically heterogeneous [78].

In non-functioning PitNETs, therapeutic management includes active surveillance, surgical resection, and radiotherapy. The neurosurgical approach is currently recommended as first-line treatment in cases with visual disturbances secondary to mass compression [79]. In tumoral recurrences after surgical excision and in tumor remnants, radiotherapy is indicated, and in asymptomatic cases, conservative treatment with surveillance is the best choice. Nevertheless, there is no specific protocol or consensus on therapeutic management regarding the timing, frequency, and duration of clinical and paraclinical assessments or regarding the management of tumor remnants or recurrences [79].

In clinically relevant PitNETs, therapeutic management includes a neurosurgical approach as first-line treatment, and prolactinomas without mass effect represent the only exception. Other treatments comprise radiation and medical therapy [80,81].

### 3.1. Exosomes as Biomarkers in PitNETs

The role of exosomes as biomarkers has already been discussed. However, their potential role as biomarkers in PitNETs has also been researched in the past few years, with promising results. In a recent study, Nemeth et al. conducted a comprehensive analysis of circulating microRNA in the plasma of patients with PitNET [82]. Given that various reports showed an abundance of microRNA in pituitary tumors, the authors wanted to analyze whether this type of nucleic acid is differentially expressed between normal pituitary tissue and tumoral tissue or in preoperative versus postoperative settings. The study concluded that a reduction of microRNA-143-3p level was exclusive to FSH/LH adenomas. At the same time, no correlations were found between the expression of this nucleic acid and tumoral size or Ki-67 index. Furthermore, the authors stated that the level decrease in this type of microRNA was specific to FSH/LH adenomas with no regard to the grade of tumor excision and did not change in patients with plurihormonal, growth-hormone secreting tumors and gonadotroph adenomas [82].

Another recent research study by Lyu et al. concluded that 18 up-regulated and 36 down-regulated microRNAs showed significant expression alterations in patients with non-functional PitNETs compared to healthy patients. The authors demonstrated that while hsa-miR-486-5p, hsa-miR-151a-5p, hsa-miR-652-3p_R+1, and hsa-miR-1180-3p are promising biomarkers for these non-functioning PitNETs, the most competent one is considered miR-486-5p [83].

Serum microRNA has also been linked to somatotrophinomas. In a recent study published by Zhao et al., 169 microRNAs were significantly expressed between somatotrophinomas and healthy hypophyses. Among these types, hsa-miR-320a and hsa-miR-423-5p had lower expression levels than healthy subjects. Moreover, preclinical models showed that microRNA-423-5p inhibited cellular proliferation, induced apoptosis, and reduced growth hormone release. Thus, the authors concluded that microRNA-423-5p has a major role in promoting tumorigenesis in somatotrophinomas [84].

In like manner, Zhang et al. investigated whether exosomal lncRNA H19 could be transported across the cellular membrane to exert its inhibitory effect on tumoral growth in PitNETs. The study not only concluded that this type of RNA indeed inhibits pituitary tumor growth but also that plasma exosomal H19 is an important biomarker for predicting medical responses in prolactinomas [85].

Chen et al. reported a new possible biomarker for invasiveness in PitNETs. The authors indicated that compared to noninvasive tumors, the invasive ones had an increased expression of exosomal N-cad, E-cad, epithelial cell adhesion molecule (Epcam), TGF-β1, and Smad3. The results suggested that the epithelial-mesenchymal transition-related biomarkers in serum exosomes could be used as potential biomarkers for invasiveness in PitNETs [86].

Circular RNAs concerning pituitary lesions were also studied. It has been stated that this type of nucleic acid is aberrantly expressed in PitNETs and is correlated to proliferation, progression, invasiveness, and secretion of growth hormone in somatotrophinomas. However, although circular RNAs are great candidates as biomarkers in PitNETs, given their stability and tissue specificity, their exact roles have yet to be fully uncovered [87,88].

### 3.2. Exosomes as a Potential Therapeutic Target in PitNETs

Circular RNAs are not only studied as biomarkers but also as therapeutic targets in pituitary lesions. Wan et al. revealed that circular MFN2 demonstrated major upregulation in aggressive PitNETs while correlating to tumoral growth, cellular transport, and morphology. According to the study, an association between the expression of this circular RNA and invasiveness and the need for surgical intervention has been observed [89]. Tumoral excision led to modifications in circular MFN2 in all patients, as the blood levels were reported as low postoperatively. Apart from investigating whether circular MFN2 could be a viable biomarker to distinguish pituitary tumors from healthy subjects, the study also discovered a novel regulatory feedback loop in PitNETs, which was constituted by circMFN2/miR-146a/TRAF6/NF-κB. Thus, the authors concluded that targeting the inhibition of circular MFN2 could be a potential treatment for PitNETs [89].

Besides the aforementioned roles of H19, it has been recently revealed that it also inhibits programmed cell death of neoplastic cells, and it has been proposed as a therapeutic target in a recent article published by Xia et al. [90].

Wu et al. demonstrated that H19 is more effective than Cabergoline in prolactinomas, and the role of the H19-mTOR-4E-BP1 axis in PitNETs growth regulation may be a potential therapeutic target [91].

Similarly, Tang et al. published a study in which they demonstrated that exosomal AFAP1-AS1 promotes tumoral growth, cellular migration, and glycolysis in PitNETs by inhibiting HuR degradation [92]. The authors suggested that targeting exosomal AFAP1-AS1 could be a promising strategy in the therapeutic management of PitNETs [92].

Rahimian et al. stated that an increased number of exosomal non-coding RNA expression profiles were discovered in PitNETs, and unveiling the expression patterns could offer not only an important source of biomarkers but also a possible therapeutic target [93].

The continuous interest in exosomes in the biomolecular field has revealed new implications in cancer and pituitary lesions. Recent findings suggest that exosomal hsa-miR-25-5p originating from somatotropinomas contributes to acromegaly, and by discovering the mechanisms behind this process, new treatment perspectives could be uncovered [94]. The study concluded that in somatotrophinomas, these specific exosomes promote bone formation and trabecula number in vitro. The process of increased trabecula formation might be associated with exosomal-induced osteoblast proliferation through increased cell viability and DNA replication [94].

Despite these findings, further studies are needed to discover the full potential of exosomal involvement in PitNETs. The possible limitations of these studies are that the majority are based on relatively small cohorts, while the assessment methods are usually different. Furthermore, not only is the isolation of exosomes without cellular contamination still very difficult, but no clinical trials evaluating the impact of these extracellular vesicles in PitNETs are available. Nevertheless, this biomolecular field has demonstrated significant potential, and future research is considered worthy [8].

### 3.3. Mechanistic Insights on the Influence of Exosomes in PitNETs

Alterations have been observed in exosomal protein expression among invasive PitNETs and non-invasive PitNETs as well. The folate receptor 1 (FOLR1) and Epcam proteins were considerably decreased in exosomes that were derived from the serum of invasive non-functional PitNETs, thereby indicating their potential role as biomarkers regarding tumor invasiveness [86,95,96]. Furthermore, these proteins were deeply connected with tumor malignancy and metastatic potential [97,98,99,100].

Exosomes contain different RNA forms, including microRNAs, long non-coding RNAs, and circular RNAs, and they change gene activity in cells that will eventually receive them. In PitNETs, some exosomal microRNAs are correlated with tumor behavior and dynamics. Furthermore, the introduction of microRNAs via exosomes stops tumor cell development and migration. Tumorigenesis modulation has involved exosomal RNAs in regard to PitNETs, as new studies have demonstrated that GH3 and MMQ rat pituitary tumor cell lines possess definitive miR-149-5p and miR-99a-3p downregulation [94,101]. These miRNAs were found to inhibit tumor cell proliferation, migration, and invasion when delivered via exosomes, suggesting their role as tumor suppressors. On a molecular level, GH3 cells derived from exosomes have been shown to stimulate, through the Smad7/Runx2 and miR-21/PDCD4/AP-1 signaling pathways, osteoblast proliferation. Moreover, they appear to exert major influence on the osteoblast differentiation when they upregulate the expression of collagen I, osteocalcin, and Runx2 [94]. These particular findings do suggest that exosomal miRNA plays a certain role in the overall development of acromegaly by disrupting the balance between osteoblast activity and osteoclast activity. This disruption can result in the abnormal formation of bone [94].

Regarding miR-21-5p, a high amount of this microRNA has been identified in exosomes originating from somatotropinomas. The main roles of miR-21-5p are to connect to greater bone cell activity and to support the acromegaly development [102].

When it comes to miR-1180, it has been stated that it can be used as an early indicator of non-functional PitNETs. In addition, its presence in exosomes suggests that it helps with early identification, thus also serving as a biomarker [103]. All of these aforementioned findings further highlight the pivotal role of exosomes and their components not only in modulating tumoral behavior but also in clinical applications.

A summary of all previously discussed research implications of exosomes in PitNETs (Section 3) is elaborated in Table 1.

## 4. Conclusions

Although the study of exosomes, especially in PitNETs, is still in its infancy, valuable findings have been reported, particularly in the last decade. These findings highlight the possibilities of using exosomes in inter-/intracellular communication, reshaping cellular metabolism, tumorigenesis, biomarkers, or targeted agents for state-of-the-art therapies. However, notwithstanding that exosomes show great promise, their clinical translation is currently limited by several obstacles such as difficulties in large-scale manufacturing, lack of standardized protocols for their isolation and purification, and issues related to precise targeting. Future perspectives and directions involve ongoing research aimed at addressing these limitations by deepening our understanding of exosome biology and developing standardized methodologies to enable their safe and effective clinical application.

## Figures and Tables

**Table 1:** A summarized rendering of biomarkers and therapeutic targets specifically linked to PitNETs.

Biomarker/Therapeutic Target	Molecular Role	Clinical Potential
**miR-149-5p**	Tumor suppressor; inhibits proliferation, migration, and invasion	Therapeutic delivery via exosomes
**miR-99a-3p**	Tumor suppressor involved in regulating tumor dynamics	Diagnostic and therapeutic use
**miR-21-5p**	Promotes osteoblast activity and contributes to acromegaly development	Biomarker for acromegaly severity
**miR-1180-3p**	Potential early diagnostic biomarker for non-functional PitNETs	Liquid biopsy candidate
**miR-143-3p**	Downregulated in FSH/LH adenomas	Subtype-specific diagnostic marker
**miR-486-5p**	Significantly dysregulated in non-functional PitNETs	High sensitivity and specificity as a diagnostic biomarker
**miR-423-5p**	Decreased in somatotrophinomas; inhibits growth hormone secretion	Therapeutic target in somatotropinomas
**lncRNA H19**	Inhibits tumoral proliferation; involved in the regulation of cellular apoptosis	Predicts medical response; therapeutic candidate
**E-cad, N-cad, Epcam**	EMT-related markers upregulated in invasive PitNETs	Serum biomarker panel for invasiveness
**circMFN2**	Promotes tumoral growth via miR-146a/TRAF6/NF-κB signaling	Potential target for aggressive PitNETs
**AFAP1-AS1**	Enhances tumoral growth, migration, and glycolysis via HuR stabilization	Targetable axis for metabolic intervention
**FOLR1**	Downregulated in invasive non-functioning PitNETs	Biomarker for tumor invasiveness
**Smad7/Runx2 Pathway**	Stimulated by GH3-derived exosomes; activates osteoblasts	Mechanistic link to bone changes in acromegaly
